# *Actinidia arguta* Sprout as a Natural Antioxidant: Ameliorating Effect on Lipopolysaccharide-Induced Cognitive Impairment

**DOI:** 10.4014/jmb.2009.09012

**Published:** 2020-10-09

**Authors:** Jeong Eun Kang, Seon Kyeong Park, Jin Yong Kang, Jong Min Kim, Bong Seok Kwon, Sang Hyun Park, Chang Jun Lee, Seul Ki Yoo, Ho Jin Heo

**Affiliations:** 1 Division of Applied Life Science, Institute of Agriculture and Life Science (BK2), Gyeongsang National University, Jinju 52828, Republic of Korea; 2 Korea Food Research Institute, Wanju-gun 55365, Republic of Korea

**Keywords:** *Actinidia arguta* sprout, antioxidants, anti-inflammation, lipopolysaccharide, neuroprotection

## Abstract

Here, we investigated the prebiotic and antioxidant effects of *Actinidia arguta* sprout water extract (AASWE) on lipopolysaccharide (LPS)-induced cognitive deficit mice. AASWE increased viable cell count, titratable acidity, and acetic acid production in *Lactobacillus reuteri* strain and showed a cytoprotective effect on LPS-induced inflammation in HT-29 cells. We assessed the behavior of LPSinduced cognitive deficit mice using Y-maze, passive avoidance and Morris water maze tests and found that administration of AASWE significantly improved learning and memory function. The AASWE group showed antioxidant activity through downregulation of malondialdehyde levels and upregulation of superoxide dismutase levels in brain tissue. In addition, the AASWE group exhibited activation of the cholinergic system with decreased acetylcholinesterase activity in brain tissue. Furthermore, AASWE effectively downregulated inflammatory mediators such as phosphorylated- JNK, phosphorylated-NF-κB, TNF-α and interleukin-6. The major bioactive compounds of AASWE were identified as quercetin-3-*O*-arabinopyranosyl(1→2)-rhamnopyranosyl(1→6)-glucopyranose, quercetin-3-*O*-apiosyl(1→2)-galactoside, rutin, and 3-caffeoylquinic acid. Based on these results, we suggest that AASWE not only increases the growth of beneficial bacteria in the intestines, but also shows an ameliorating effect on LPS-induced cognitive impairment.

## Introduction

The human microbiome is a collection of all microbial groups naturally present in the human body and plays an important role in maintaining the mucosal immune system and normal intestinal physiology [1]. Intestinal microorganisms and their metabolites have been recently found to affect not only intestinal but also overall host functions. The imbalance of intestinal microorganisms is linked to various diseases such as obesity, diabetes, metabolic syndrome, inflammatory bowel disease, and autoimmune disease [2]. In particular, the connection between intestinal and neuronal function is attracting attention [3, 4]. Western and other high-fat diets cause intestinal microflora imbalance and reduce metabolites such as short-chain fatty acids (SCFAs). They can also promote the growth of gram-negative bacteria that damage the intestinal wall and produce endotoxins. Furthermore, intestinal gram-negative bacteria can release inflammatory cytokines, causing hippocampal dysfunction and blood brain barrier (BBB) damage, leading to impaired cognitive function [4]. The use of prebiotics that beneficially alter intestinal microorganisms can help overcome an imbalance of intestinal microorganisms and potentially improve metabolic syndrome. Recent studies have reported that prebiotic intake leads to significant changes in various disease models by exerting anti-inflammatory and neurophysiological activities [5, 6]. *Lactobacillus reuteri* are representative lactic acid bacteria naturally present in the human body. In addition, *L. reuteri* can interact with other beneficial bacteria to create a balanced, steady state in the gastrointestinal immune response and contribute to the prevention of inflammation while improving and maintaining intestinal integrity and function [7]. In particular, *L. reuteri*, a potential probiotic known to modulate the immune system, has been reported to affect microbiota-brain interactions [8]. *L. reuteri* has been shown to improve anxiety and depression-like behavior by altering the mRNA expression of γ-aminobutyric acid receptors in the central nervous system of mice [8].

Lipopolysaccharide (LPS), an outer membrane component of gram-negative bacteria, is composed of a lipophilic group lipid A and a hydrophilic group polysaccharide. Intraperitoneal injection of LPS induces a nonspecific immune response to activate B cells and stimulates macrophages to secrete cytokines such as interleukin-1β (IL-1β), interleukin-6 (IL-6), and TNF-alpha (TNF-α). Moreover, LPS-induced inflammation accumulates Aβ through changes in β-secretase and γ-secretase activity and results in a gradual loss of dopaminergic neurons in the substantia nigra [9]. In addition, systemic injection of LPS increases the intracerebral inflow of blood Aβ through alterations in lipoprotein receptor-related protein-1, which is the brain-to-blood transporter of Aβ, and can ultimately lead to cognitive defects [9].

*Actinidia arguta* is a perennial vine plant cultivated in Korea, Japan and China. It has been used as a traditional medicine due to its various nutrients and physiologically active substances [10]. Our previous study showed that the ethyl acetate fraction of *A. arguta* improves trimethyltin-induced cognitive deficits due to various phenolic compounds [11]. According to Kim *et al*. [12], the chloroform fraction of *A. arguta* stem improved inflammation through inhibition of the NF-κB pathway in macrophages. In addition, *A. arguta* sprout is reported to improve postprandial blood glucose levels by inhibiting α-glucosidase in streptozotocin-induced diabetic rats [13]. However, there is a lack of research on the effect of *A. arguta* on the relationship between intestinal microorganisms and cognitive function. *L. reuteri*, a representative beneficial lactic acid bacterium in the human body, has been reported to have anti-obesity effects and an ameliorating activity on LPS-induced inflammation, and to affect microbiota-brain interaction [8, 14]. Therefore, in this study we assessed the effect of *A. arguta* on the growth of probiotic strains (*L. reuteri*) in vitro and examined the ameliorating activity on an LPS-induced neuroinflammation model.

## Materials and Methods

### Materials

De Man-Rogosa-Sharp (MRS) broth and fructo-oligosaccharide (FOS) were purchased from Difco (USA) and Wako (Japan), respectively. A superoxide dismutase (SOD) kit was obtained from Dojindo Molecular Technologies (USA). Anti-*p*-JNK (sc-6254) and anti-β-actin (sc-69879) were purchased from Santa Cruz Biotechnology (USA). Anti-*p*-NF-κB (3033S) and anti-TNF-α (3707S) and secondary antibodies were purchased from Cell Signaling Technology (USA). Anti-IL-6 (P05231) was obtained from Cusabio Biotech (China). A mouse IL-6 enzyme-linked immunosorbent assay (ELISA) kit was purchased from Koma Biotech (Korea). All other products including LPS (*Escherichia coli* O55: B5), RPMI 1640 medium, HEPES, metaphosphoric acid and sodium bicarbonate were purchased from Sigma-Aldrich Chemical Co. (USA).

### Sample Preparation

*A. arguta* sprout (cultivar: Autumn Sense) was purchased from Sansarang (Korea) which collected it from a mountain valley in Korea in May 2017. *A. arguta* sprout was extracted in distilled water at 40°C for 2 h, and the obtained water extract was filtered and concentrated using a rotary evaporator. Then, the water extract of *A. arguta* sprout was lyophilized and stored at -20°C for later use.

### Measurement of Growth

**Probiotic strain and growth conditions.** *L. reuteri* strain was obtained from the Korea Collection for Type Cultures (Korea). The KCTC 3594 strain of *L. reuteri* was isolated from the intestines of an adult and cultured in MRS broth.

**Determination of growth rate.** Assays were carried out in MRS broth (supplemented with 0.5 g/l of L-cysteine/HCl) containing 0.1% (w/v) glucose, which was used as a control medium. The FOS and AASWE groups evaluated *L. reuteri* using culture medium supplemented with 2% (w/v) FOS or 2% (w/v) AASWE as a carbon source in the control group medium. After inoculation, the bacteria were incubated for 48 h at 37°C. Samples (*n* = 3) were taken at several time periods, plated in MRS agar by the spread technique and incubated at 37°C for 48 h. Viable cells were measured by colony count, and appeared as log CFU/ml [15].

### Determination of Titratable Acidity

Pre-treatment of titratable acidity measurement was the same as that of viable cell count with 1% (v/v) phenolphthalein as an indicator. The culture media was titrated with 0.1 N (w/v) NaOH solution until the sample turned red. The optimum consumption was measured, and total acid in the culture media was converted into lactic acid.

### Determination of Acetic Acid Production

*L. reuteri* supernatant (*n* = 3) containing an internal standard (2-ethylbutyric acid) was mixed, and propanol/pyridine mixture solvent (v/v = 3:2) and propylchloroformate were subsequently added. After vortexing, derivatization was performed via sonication for 1 min. Hexane was added to the reaction mixture that was then centrifuged at 14,000 ×*g*. The upper hexane layer was transferred to another tube and extracted one more time. The extracted hexane layer was used as an acetic acid extract. In order to measure SCFA production in *L. reuteri*, GC-MS analysis was carried out using an GC-MS-TQ 8030 triple quadrupole mass spectrometer (Shimadzu, Japan), GC/MS-QP 2010 Plus and DB-5MS column (5% phenyl methyl siloxane, 30 m × 0.25 mm, thickness 0.25 μm). The GC conditions were as follows: split ratio 100:1, injection temp 200°C, column oven temp 60°C, with and flow rate of 1.0 ml/min. The initial temperature was 60°C and maintained for 2 min, then from 70 to 85°C at a rate of 10°C /min, and then from 85 to 110°C at a rate of 4°C /min, then finally from 110 to 290°C at a rate of 90°C /min, and maintained for 6 min.

### Cell Culture and Viability Assay

An HT-29 cell line was received from Korea Cell Line Bank (Korea) and grown in RPMI 1640 medium including 10% fetal bovine serum, 25 mM sodium bicarbonate, 25 mM HEPES and 1% antibiotics. HT-29 cells were plated on 96-well plates at a density of 1 × 10^4^ cells/well for 24 h. Seeded cells were treated with FOS or various concentrations of AASWE (*n* = 3) for 30 min, and then LPS was added. After 24 h incubation, MTT stock solution was reacted for 3 h. Media were removed, and the produced MTT formazan crystals were dissolved using DMSO. The formed formazan was measured at 570 nm using a microplate reader (Epoch 2; BioTek Instruments, Inc., USA).

### Animal Experiment

All experimental procedures were approved by guidelines established by the Institutional Animal Care and Use Committee of Gyeongsang National University (Certificate No. GNU-170727-M0035) on July 27, 2017. Four-week-old male, Institute of Cancer Research (ICR) mice were purchased from Samtako (Korea). The mice were randomly assigned three per cage, were freely fed fodder and water, and bred under conditions of 12 h light-dark cycle, 55% humidity, and 22 ± 2°C. The mice were divided into six groups (*n* = 7), which consisted of a control group, LPS-injected group (negative control group), and AASWE intake groups (20 and 50 mg/kg of body weight, respectively). The AASWE was dissolved in drinking water and orally administered for 3 weeks. After 3 weeks, LPS (final concentration of 0.1 mg/ml) was intraperitoneally injected daily for 7 days at 500 μg/kg of body weight.

### Y-Maze Test

Behavioral experiments were performed after LPS injection for 7 days. The maze test was carried out in a black plastic Y-shaped maze (33 cm long, 15 cm high, and 10 cm wide), and each mouse (*n* = 7 per group) was placed on the tip of one arm and permitted to move freely for 8 min. The movements of each mouse were recorded and evaluated using a SMART video tracking system (SMART v3.0; Panlab SL, Spain). Alternation behavior was computed as a percentage of the number of times that all three arms were entered.

### Passive Avoidance Test

A passive avoidance test was performed to investigate short-term learning and memory ability. The test chamber consisted of light and dark chambers. Each mouse (*n* = 7 per group) was acclimated for 1 min in the lighted chamber, and electric shock (0.5 mA, 3 s) was given when the door was opened and they entered the dark chamber. After 1 day, each mouse was located in the lighted chamber and the latency time to enter the dark chamber was measured for a maximum of 300 s.

### Morris Water Maze Test

A Morris water maze test was conducted using a stainless-steel circular pool (90 cm in diameter). The movements and latency time of each mouse were recorded and evaluated during the test using the SMART video tracking system. The pool water was filled with squid ink (Cebesa, Spain) to hide the platform (6 cm in diameter) as an escape place in the center of N zone. Training (days 1-4) was conducted four times a day, and the escape latency time for each mouse (*n* = 7 per group) to go to the platform was measured for 60 s. If a mouse did not reach the platform within 60 s, it was guided to the platform and left there for 15 s. For the probe test (day 5), the platform was removed and the stay time of each mouse in the N zone was measured for 90 s.

### Antioxidant Effect of AASWE in Brain Tissue

**Determination of MDA content.** After the behavioral tests, blood was drawn from the abdominal aorta. The brain tissue was immediately isolated for biochemical analysis and washed with ice-cold phosphate-buffered saline (PBS; pH7.4; 137 mM NaCl, 2.7 mM KCl, 4.3 mM Na_2_HPO_3_, and 1.4 mM KH_2_PO_4_) and kept at −80°C until use.

To measure MDA levels, brain tissues homogenized with 10 volumes of PBS were centrifuged at 2,450 ×*g* for 10 min at 4°C (*n* = 7 per group). Supernatant was mixed with 1% (v/v) phosphoric acid, and 0.67% (v/v) thiobarbituric acid solution was added and reacted at 95°C for 1 h. After cooling, the reaction product was centrifuged and a supernatant, which was measured at 532 nm using a spectrophotometer (Shimadzu UV-1601; Japan), was obtained.

**Determination of SOD content.** To determine the SOD content, 800 μl of cold PBS was added to 200 μl of homogenated tissue and centrifuged to obtain a pellet (*n* = 7 per group). Cell extraction buffer was added and then stored on ice for 30 min. After incubation, the extract was centrifuged at 10,000 ×*g* for 10 min, and the supernatant was used for the experiment. SOD activity was measured using an SOD assay kit following the manufacturer’s instructions. The measured SOD activity values were calculated with a standard curve and converted to SOD level (U/ml).

### Determination of Cholinergic System

**Determination of acetylcholine (ACh) content.** To measure ACh content and acetylcholinesterase (AChE) activity, the brain homogenate was centrifuged at 14,000 ×*g* for 30 min at 4°C and the supernatant was used (*n* =7 per group). To measure the ACh content, the supernatant and alkaline hydroxylamine reagent were mixed at room temperature for 1 min, and then 0.5 N hydrochloride and 0.3 M iron (III) chloride hexahydrate were added.

**Determination of AChE activity.** To measure AChE activity, supernatant (*n* = 7 per group) and 50 mM sodium phosphate buffer (pH 7.4) were reacted at 37°C for 15 min. Then, 500 μM AChE substrate solution was added and reacted at 37°C for 10 min. The results were evaluated for 10 min using a microplate reader.

### Western Blot Assay

Brain tissues (*n* = 5 per group) were homogenized with ProtinEx Animal cell/tissue (Gene All Biotechnology, Korea) containing 1% protease inhibitor cocktails (Thermo Fisher Scientific, USA). After centrifugation (13,000 ×*g*, 10 min, 4°C), the supernatant was quantified by Bradford reagent (Bio-Rad, USA), and boiled with Laemelli buffer (5X) at 95°C for 7 min. Protein was separated on a polyacrylamide gel and transferred to a poly-vinylidene difluoride membrane. After transfer, the membrane was blocked with 5% skim milk and then incubated overnight in TBS containing 0.1% Tween-20 (TBST) including each diluted primary antibody (1:1,000) at 4°C. The membrane was then reacted with secondary antibody for 1 h at room temperature. For detection, immune complexes were visualized using tetramethylbenzidine (TMB) reagent as substrate and the ProNA ECL Ottimo (TransLab, Korea) using an iBright CL1000 Imaging System (Thermo Fischer Scientific).

### Determination of IL-6 Content

The IL-6 level in brain tissue was measured using an ELISA kit following the manufacturer's instructions. In brief, the supernatant prepared for western blot assay was diluted 10-fold using a dilution assay solution and then used as a sample for ELISA (*n* = 5). The sample and biotinylated antibody were added to a pre-coated 96-well plate with anti-mouse IL-6 antibody for 2 h at room temperature. For detection, TMB substrate solution was added, and the reaction was terminated by TMB stop solution. The absorbance was measured at 450 nm with a microplate reader (Epoch2; BioTek, USA).

### Identification of Bioactive Compounds

Ultra-performance liquid chromatography (UPLC) accurate-mass quadrupole time-of-flight (Q-TOF)/MS (Acquity UPLC Class 1; Waters Corp., USA) and an Acquity UPLC BEH C18 column (2.1 × 100 mm, 1.7 μm particle size; Waters Corp.) were used for the main phenolic compound analysis of AASWE. The flow rate was 0.35 ml/min, and oven temperature was 40°C. The gradient program was 0.1% B (99.9% A) to 25% B (75% A) at 0-2.0 min, to 55% B (45% A) at 2.0-8.0 min using solvent A (0.1% formic acid in distilled water) and solvent B (0.1%formic acid in acetonitrile). MS conditions were as follows: negative-ion mode, drying gas (N2) heated to 120°C, and collision energy at 20-40V.

### Statistical Analysis

All results were shown as means ± standard deviation (SD). The significance distribution of difference between groups was determined by one-way analysis of variance (ANOVA) followed by a Duncan’s multiple range test with SAS ver. 9.1 (SAS Institute Inc., USA).

## Results

### Effect of AASWE on Growth and Activity of *L. reuteri*

**Fig. 1:**
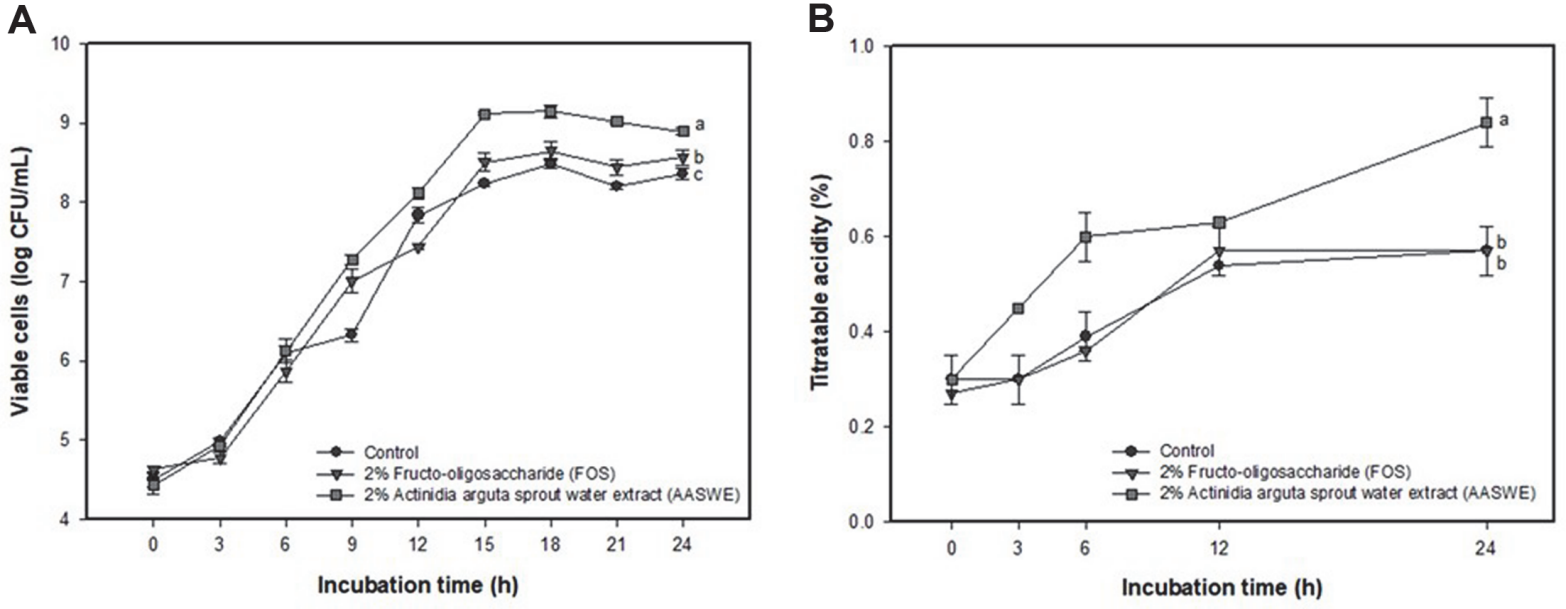
Effect of *Actinidia arguta* sprout water extract (AASWE) on viable cell count (A) and titratable acidity measurement (B) in *Lactobacillus reuteri* strain. Data were analyzed using ANOVA with Duncan’s SAS and expressed as mean ± SD (*n* = 3). Each small letter shows statistical difference and was statistically considered at *p* < 0.05.

The measurement of viable cell count and titratable acidity using *L. reuteri* strain are shown in Fig. 1. As time passed, the viable cells of *L. reuteri* tended to increase in all groups (Fig. 1A). The AASWE-treated strain showed a relatively higher viable count (6.12 ± 0.15 log CFU/ml) compared to the FOS-treated strain (5.87 ± 0.14 log CFU/ml) as a positive control after 6 h incubation, and indicated the highest number of viable cells at 18 h incubation (9.14± 0.09 log CFU/ml). The highest titratable acidity value was shown in the AASWE-treated strain (0.84 ± 0.05%)(Fig. 1B). Then, the quantitative values of acetic acid were measured, and the results are shown in Table 1. Acetic acid content was about two times higher in the AASWE-treated strain (106.55 ± 26.18 mM) than in the control (50.36 ± 5.38 mM) and FOS-treated strain (55.50 ± 1.31 mM).

### Effect of AASWE on LPS-Induced HT-29 Cells

**Fig. 2:**
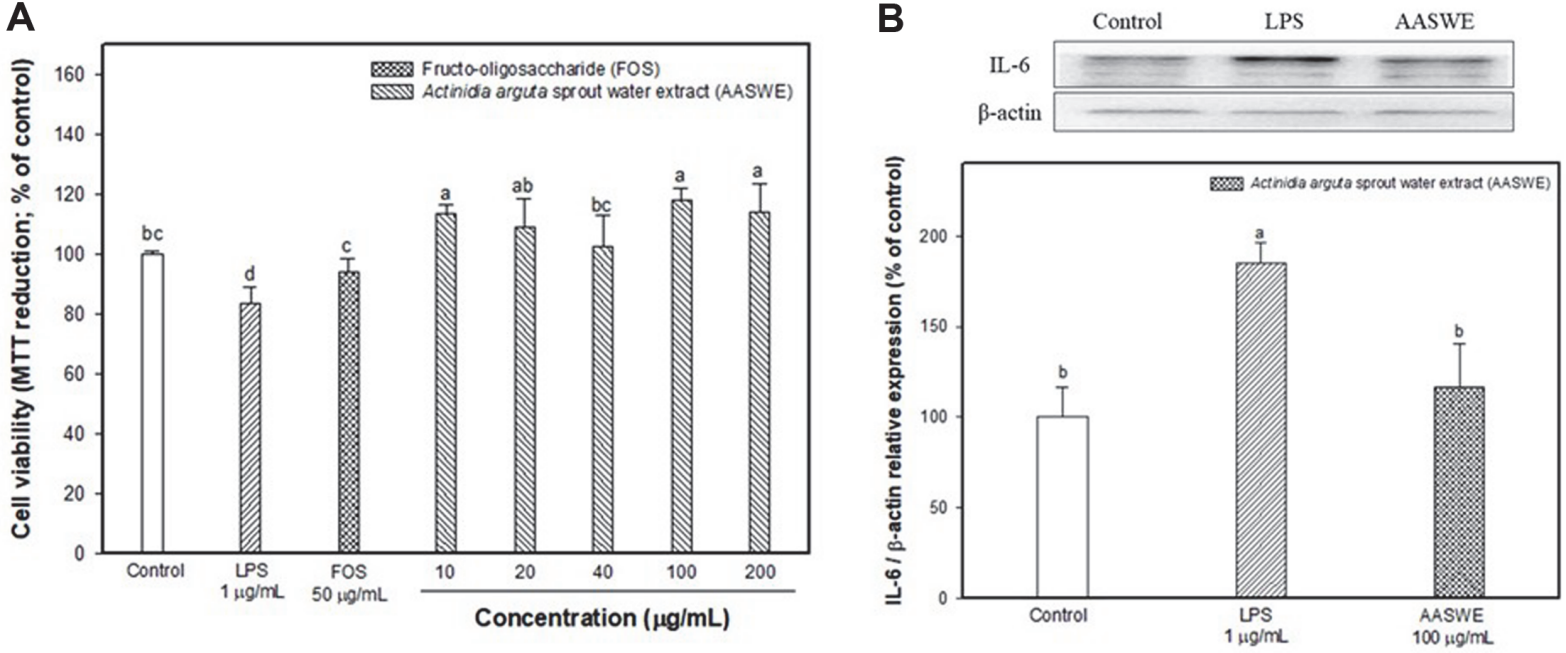
Effect of *Actinidia arguta* sprout water extract (AASWE) on LPS-induced cognitive impairment mice. Effect of *Actinidia arguta* sprout water extract (AASWE) on cell viability (**A**) and the protein expression levels of interleukin-6 (**B**) on LPS-induced HT-29 cell. Data were analyzed using ANOVA with Duncan’s SAS and expressed as mean ± SD (*n* = 3). Each small letter shows statistical difference and was statistically considered at *p* < 0.05.

The protective effect of AASWE on LPS-induced intestinal inflammation in HT-29 cells is shown in Fig. 2. The LPS-treated cells showed decreased cell viability (83.62 ± 5.60%), and the FOS-treated cells (93.99 ± 4.82%) exhibited significant improvement at 50 μg/ml concentration compared with the control group (100.00 ± 1.25%)(Fig. 2A). On the other hand, AASWE-treated cells effectively protected against LPS-induced cytotoxicity at a concentration of 10 μg/ml or more. IL-6 levels were measured to confirm an ameliorating effect on LPS-induced inflammation in HT-29 cells, and LPS-treated cells indicated an increased IL-6 level of about two times (Fig. 2B). AASWE-treated cells effectively inhibited the IL-6 level at a concentration of 100 μg/ml.

### Effect of AASWE on Behavior

**Fig. 3:**
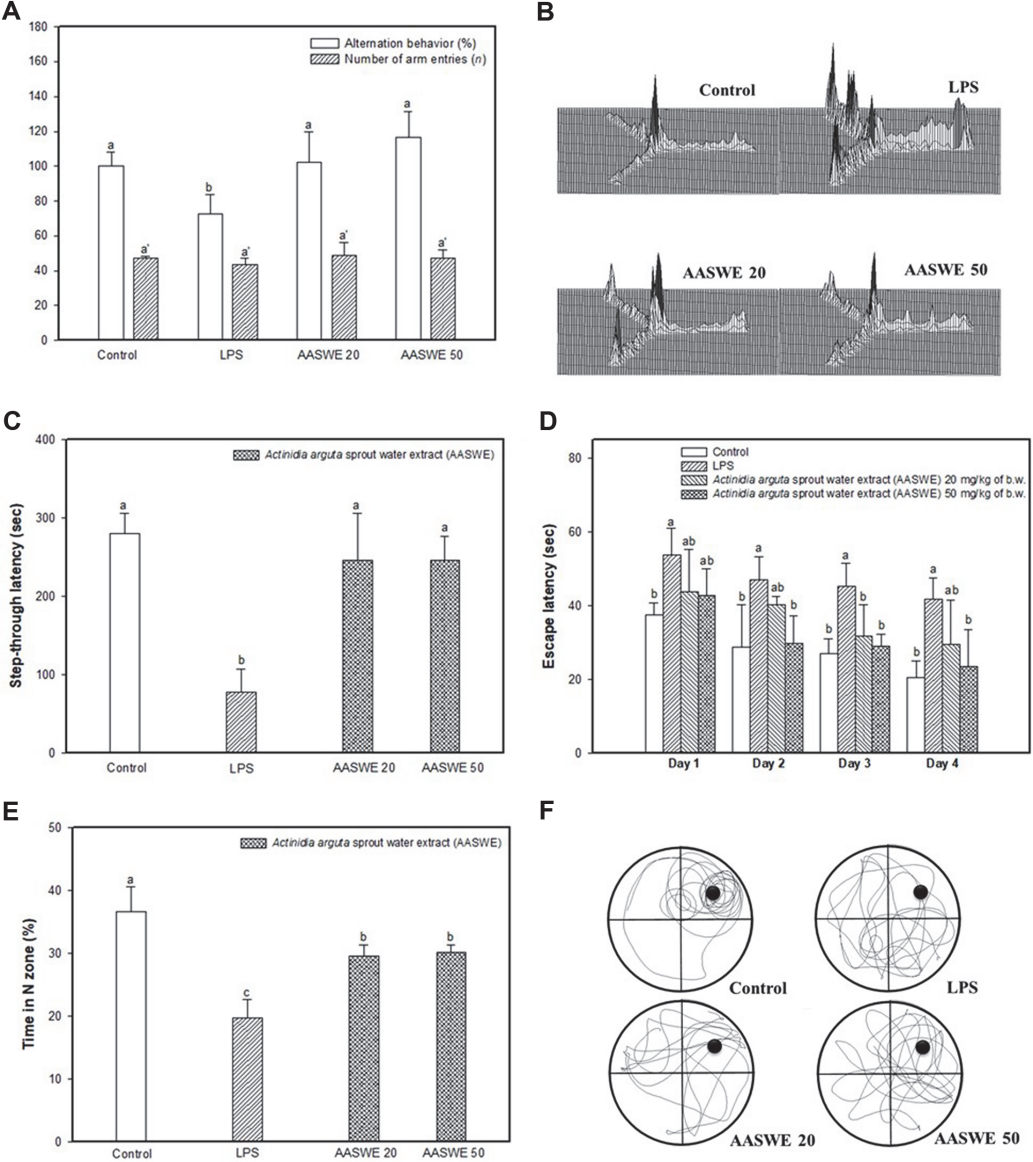
Effect of *Actinidia arguta* sprout water extract (AASWE) on LPS-induced cognitive impairment mice. Alteration behavior and number of arm entries (**A**), the path tracing of each group in the Y-maze test (**B**), step-through latency in passive avoidance test (**C**), escape latency in the training trial (**D**), time in N zone in the probe test (**E**), and the path of motion in the probe test (**F**). Data were analyzed using ANOVA with Duncan’s SAS and expressed as mean ± SD (*n* = 7). Each small letter shows statistical difference and was statistically considered at *p* < 0.05.

To measure spatial learning memory, a Y-maze test was conducted (Figs. 3A and 3B). The number of arm entries was statistically similar in all groups, and alternation behavior showed that the LPS group (72.78 ± 11.06%) decreased compared with the control group (100.00 ± 8.31%) (Fig. 3A). However, the administration of AASWE effectively improved the alternation behavior (AASWE 20; 102.20 ± 17.43% and AASWE 50; 116.61 ± 14.80%, respectively) compared with the LPS group. The representative movement routes of each group are shown in Fig. 3B. The movement routes of the LPS group were irregular in comparison with the control group, and the AASWE group appeared to have similar tendencies to the control group.

To confirm the improvement of short-term memory impairment with AASWE, passive avoidance tests were conducted, and the results are shown in Fig. 3C. The step-through latency of the LPS group decreased (78.25 ± 28.55 s) compared to the control group (279.75 ± 26.59 s), and the AASWE 20 (246.00 ± 60.02 s) and 50 groups (246.00 ± 31.21 s) showed similar trends to the control group.

The results of the Morris water maze test, which measures spatial learning and long-term memory, are shown in Figs. 3D-3F. As the training progressed, the time to find the hidden platform decreased in all groups (Fig. 3D). On day 4 in the hidden platform test, the AASWE group had lower escape latency times than the LPS group. After the training periods, a probe test was performed to measure the time to stay in the target zone (N zone) after removing the platform (Fig. 3E) The LPS group showed a lower retention time in the N zone (19.81 ± 2.91%) than the control group (36.60 ± 3.98%). However, the AASWE 20 and 50 groups showed increased retention times of 29.64 ± 1.78%and 30.14 ± 1.25%, respectively. Additionally, the mouse tracing path indicated that the LPS group stayed shorter in the N zone than the control group (Fig. 3F). However, the AASWE group increased the rate of stay in the target zone.

### Antioxidant Effect of AASWE in Brain Tissue

**Fig. 4:**
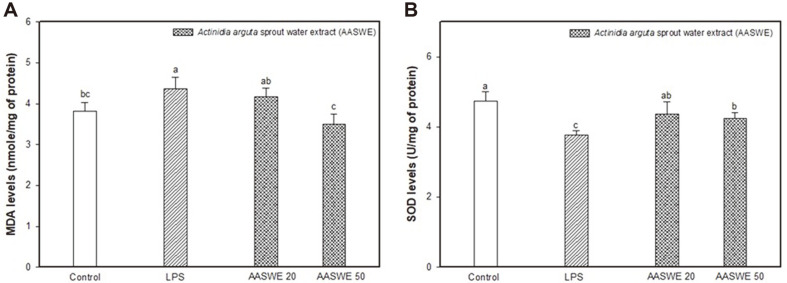
Effect of *Actinidia arguta* sprout water extract (AASWE) on MDA (A) and SOD levels (B) in LPSinduced cognitive impairment mice brain tissues. Data were analyzed using ANOVA with Duncan’s SAS and expressed as mean ± SD (*n* = 7). Each small letter shows statistical difference and was statistically considered at *p* < 0.05.

The antioxidant effect of AASWE on LPS-induced oxidative stress in mouse brain tissue was measured using MDA and SOD levels (Fig. 4). In the LPS group, the MDA level increased to 4.37 ± 0.28 nmole/mg of protein compared to the control group (3.82 ± 0.21 nmole/mg of protein), whereas it decreased to 4.17 ± 0.21 nmole/mg of protein and 3.50 ± 0.25 nmole/mg of protein in the AASWE 20 and 50 groups, respectively (Fig. 4A). SOD levels in the LPS group decreased (3.77 ± 0.13 U/mg of protein) in comparison to the control group (4.73 ± 0.29 U/mg of protein) (Fig. 4B). On the other hand, the administration of AASWE increased the SOD level at AASWE 20 (4.38± 0.34 U/mg of protein) and 50 mg/kg of body weight (4.24 ± 0.18 U/mg of protein) concentration.

### Effect of AASWE on Cholinergic System

**Fig. 5:**
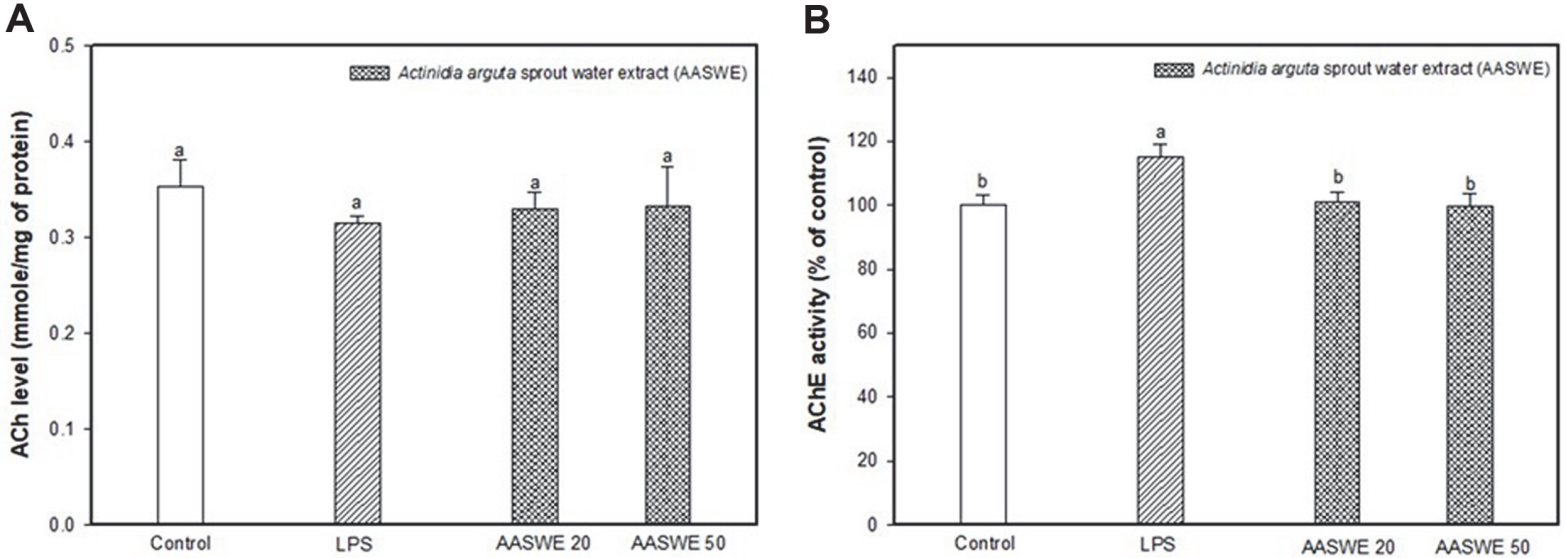
Effect of *Actinidia arguta* sprout water extract (AASWE) on cholinergic system in LPS-induced cognitive impairment mice brain tissues. The levels of ACh (**A**) and activity of AChE (**B**). Data were analyzed using ANOVA with Duncan’s SAS and expressed as mean ± SD (*n* = 7). Each small letter shows statistical difference and was statistically considered at *p* < 0.05.

As shown in Fig. 5A, ACh levels were not significantly different in all groups. However, the LPS group showed higher AChE activity (115.10 ± 4.10%) than the control group (100.00 ± 3.19%) (Fig. 5B). The AASWE 20 and 50 groups (101.25 ± 2.75% and 99.55 ± 4.06%, respectively) had statistically decreased AChE activity compared with the LPS group.

### Effect of AASWE on LPS-Induced Neuroinflammation

**Fig. 6:**
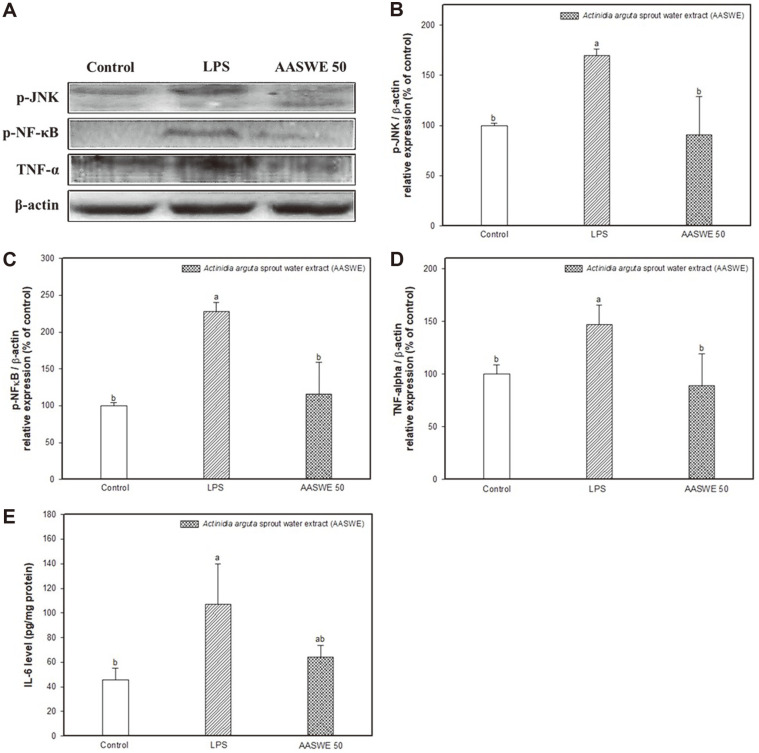
Effect of *Actinidia arguta* sprout water extract (AASWE) on inflammatory pathway. Band images (**A**), the expression levels of *p*-JNK (**B**), *p*-NF-κB (**C**), TNF-α (**D**) by western blot, and IL-6 level (**E**) by ELISA kit on LPS-induced cognitive impairment mice brain tissues. Data were analyzed using ANOVA with Duncan’s SAS and expressed as mean ± SD (*n* = 5). Each small letter shows statistical difference and was statistically considered at *p* < 0.05.

To evaluate the inhibitory effect of AASWE on LPS-induced inflammation in brain tissue, the inflammatory-mediated proteins were measured by western blot (*p*-JNK, *p*-NF-κB and TNF-α) and ELISA assay (IL-6) (Fig. 6).

The expression level of *p*-JNK increased in the LPS group (169.69 ± 6.63%) compared with the control group (100.00 ± 2.28%), and the AASWE 50 group decreased (90.61 ± 38.05%) compared with the LPS group (Figs. 6A and 6B). The expression level of *p*-NF-κB increased by LPS treatment (228.03 ± 12.31%) compared with the control group (100.00 ± 4.24%), and its inflammatory response was controlled by the administration of AASWE (116.07 ± 42.85%) (Figs. 6A and 6C). As a result of inflammatory response, pro-inflammatory cytokines (TNF-α and IL-6) were measured. TNF-α was significantly increased in the LPS group (147.17 ± 18.725%) compared with the control group (100.00 ± 9.15%) (Figs. 6A and 6D). On the other hand, the administration of AASWE (89.21 ± 30.31%) effectively inhibited pro-inflammatory cytokines. In addition, IL-6 contents increased by LPS treatment (106.92 ± 32.86 pg/mg protein) compared with the control group (45.38 ± 9.61 pg/mg protein), and its pro-inflammatory cytokines decreased with the administration of AASWE (63.97 ± 9.68 pg/mg protein) (Fig. 6E).

### Identification of Major Bioactive Compounds

**Fig. 7:**
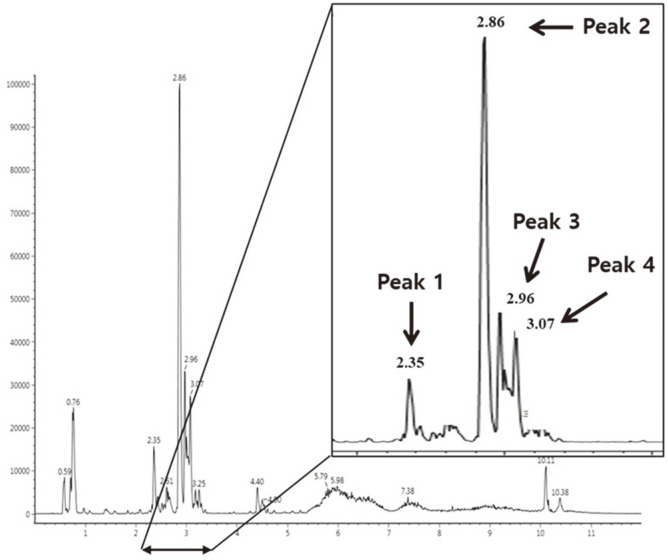
UPLC-QTOF/MS spectra of *Actinidia arguta* sprout water extract (AASWE).

The major bioactive substances of AASWE were analyzed using a UPLC-QTOF/MS^2^ system in ESI-negative ion mode (Fig. 7). The main compounds were identified by comparing the main fragments of MS^2^ scans: 3-*O*-caffeoylquinic acid (RT: 2.35 min, *m/z* 353.08; 135.04, 179.03, and 191.05); quercetin-3-*O*-arabinopyranosyl(1→2)-rhamnopyranosyl(1→6)-glucopyranose (RT: 2.86 min, *m/z* 741.18; 255.03, 271.02, and 300.02); quercetin-3-*O*-apiosyl(1→2)-galactoside (RT: 2.96 min, *m/z* 595.13; 255.03, 271.02, 300.02, and 301.03); and rutin (RT: 3.07 min, *m/z* 609.14; 255.03, 271.02, and 300.02) [16-19].

## Discussion

The term “microbiome-gut-brain axis” was coined recently, and it has been found that intestinal microorganisms affect neurophysiology, including mood and behavior as well as brain development [3]. The administration of certain probiotic species such as *Bifidobacteria* and *Lactobacillus* alters the neurotrophic molecule or neurotransmitter system and has anxiolytic- and antidepressant-like agents [20, 21]. It has also been reported that a wide range of probiotics exhibit anti-inflammatory activity and certain probiotics inhibit the rise of stress-induced plasma corticosterone [22]. Our results showed that AASWE as a prebiotic material statistically influenced the high growth rate, titratable acidity and acetic acid production of *L. reuteri* strain (Fig. 1). Recently, Parkar *et al*. [23] reported that various varieties of kiwifruit containing fibers and polyphenols show prebiotic activity by controlling intestinal microbial metabolism. These kiwifruit varieties increased the relative amounts of the beneficial bacteria *Bifidobacterium* and *Ruminososaceae* [23]. AASWE containing bioactive substances demonstrates prebiotic potential by stimulating the growth of lactic acid bacteria strains.

LPS induces Toll-like receptor 4 (TLR4) expression in colon mucosa and epithelial cells, causing colonic inflammation and damaging the intestinal epithelium. Recently, various probiotics and prebiotics have been shown to inhibit intestinal inflammation [24, 25]. Duary *et al*. [24] reported that the indigenous probiotic strains *L. plantarum* Lp9 and Lp91 exhibited proper immunomodulatory and anti-inflammatory functions induced by LPS in HT-29 cells. Also, germinal barley foodstuff as a prebiotic effectively inhibited colon inflammation by controlling inflammatory factors such as NF-κB and IL-6 [25]. Based on these reports, AASWE as a prebiotic may be a useful substance in intestinal inflammatory response by increasing cell viability and decreasing the IL-6 level of LPS-induced cytotoxicity in HT-29 cells (Fig. 2). Harmful bacteria-derived LPS causes intestinal permeability and leads to an inflammatory response and metabolic syndrome by entering the blood [4]. The inflow of LPS increases inflammatory genes such as inducible nitric oxide synthase (iNOS), cyclooxygenase-2 (COX-2), and glial fibrillary acidic protein, while also increasing the accumulation of Aβ by elevating β-secretase and γ-secretase activity. The accumulation of Aβ changes the synaptic structure and function of the hippocampus and ultimately induces cognitive deficits [9]. Our results also showed that LPS injection induced cognitive deficits in ICR mice, and the administration of AASWE effectively ameliorated learning and memory function deficits in Y-maze, passive avoidance and Morris water maze tests (Fig. 3). Recently, probiotics and prebiotics have been reported to alter brain neurochemistry through host-microbial interactions and affect cognitive function [26]. A_β1-42_-induced Alzheimer’s disease (AD) mice showed significantly improved cognitive deficits after 2 weeks of chitosan oligosaccharides (COS) administration as prebiotics in the passive avoidance and Morris water maze tests [5]. In the D-galactose-induced AD mice model, the administration of FOS enhanced spatial learning and memory capacity in the Morris water maze test. It was reported that the hydrogen gas produced in the colon by the fermentation of FOS reduced oxidative stress and played an antioxidant role in alleviating the development of AD [6]. In addition, chronic treatment with flavonoids regulated inflammatory response by decreasing the COX-2 and iNOS levels in an LPS-induced cognitive deficit model [27].

High levels of reactive oxygen species (ROS) lead to oxidative damages, which is known to be one of the major factors mediating behavioral and memory deficits. MDA is a result of fipid peroxidation due to excessive oxidative stress in the cell and thus may be an indicator of oxidative stress. Antioxidant enzymes like SOD, catalase (CAT) and glutathione peroxidase (GPx) have been known to protect cells against various diseases. SOD catalyzes the switching of intracellular reactive oxygen to hydrogen peroxide (H_2_O_2_), and the resulting H_2_O_2_ is converted to water and oxygen by CAT or GPx [28]. In our results, the administration of AASWE protected brain tissue from LPS-induced oxidative stress by decreasing the MDA content and increasing the SOD content (Fig. 4). The increasing fecal *Bifidobacteria* by FOS uptake showed reduced plasma lipid peroxidation and a free radical scavenging effect by reducing systemic oxidative damage with the colonization of colon bacteria [29].

The cholinergic system plays a crucial role in assessing cognitive functions such as learning and memory. In our results, administration of AASWE effectively decreased AChE activity compared with LPS injection (Fig. 5). LPS is known to increase pro-inflammatory cytokines and AChE activity, which leads to neurodegenerative diseases. ACh, one of the major parasympathetic neurotransmitters, suppresses LPS-induced pro-inflammatory cytokines production (IL-1 and TNF-α). Levels of ACh are constantly regulated by the hydrolytic enzyme AChE, which quickly decreases ACh in the brain [30]. Tyagi *et al*. [30] reported that LPS-induced neuronal inflammation increases the activity of AChE. And various kiwifruit varieties have been reported to have AChE inhibitory activity in in vitro, and their results were highly correlated with total phenolic contents [31].

TLR4 plays an essential role in LPS-induced signal transduction as a major receptor, and the mitogen-activated protein kinase (MAPK) pathway including JNK, ERK and p38 is activated through interaction with myeloid differentiation factor 88 and NF-κB, which plays a central role in inflammation by inducing the transcription of inflammatory genes. The activity of NF-κB is inhibited by forming a complex with the IκB protein in the cytoplasm, but when IκB is phosphorylated, the NF-κB dimer is transferred to the nucleus to activate proinflammatory cytokines such as TNF-α and IL-6 [12].

In addition, LPS is known to directly stimulate the production of pro-inflammatory cytokines such as IL-6, TNF-α and IL-1β in peripheral organs [32, 33]. These cytokines are circulated through the blood and cause systemic inflammation in various organs. In particular, the cytokines can pass to BBB and affect inflammation within the brain by activating the microglia and induce another source of cytokines [33, 34]. The increased inflammatory factors induce neuroinflammation, and then cause inflammatory brain diseases [9]. Therefore, the inhibition of pro-inflammatory factors is important for preventing inflammation-related cognitive decline. Although the influence of cytokines in the blood cannot be clearly controlled in the brain because blood cytokines were not removed, we confirmed an increase of cytokines (TNF-α and IL-6) in the brain by LPS injection. According to Savignac *et al*. [35], the administration of prebiotics ameliorated inflammatory anxiety by reducing pro-inflammatory factors such as IL-1 and serotonin 2A receptor in LPS-induced mice. This indicates that prebiotics have a potential role in the treatment of neuropsychiatric disorders characterized by anxiety and neuroinflammation [35]. Also, prebiotics increase the number of beneficial bacteria in the intestines and affect the immune system through changes in cytokine expression [36]. In addition, COS as major prebiotics significantly inhibited the phosphorylation of ERK, JNK, and p38 MAPK by significantly inhibiting the binding of LPS to the TLR4 receptor in LPS-induced RAW264.7 cells. Moreover, the COS-reduced NF-κB pathway significantly decreased inflammatory mediators such as IL-1β and NO [37]. Therefore, prebiotic materials affect not only the growth and activity of beneficial bacteria in the intestines but also the immune system through the regulation of inflammation.

In our study, the major bioactive compounds of AASWE were identified as phenolics such as quercetin-3-*O*-arabinopyranosyl(1→2)-rhamnopyranosyl(1→6)-glucopyranose, quercetin-3-*O*-apiosyl(1→2)-galactoside, rutin, and 3-*O*-caffeoylquinic acid (Fig. 7). Flavonoids showed anti-inflammatory activity both in vitro and in vivo, and in particular, quercetin inhibited iNOS and TNF-α expression by inhibiting p38 and JNK [38]. Hou *et al*. [39] reported that the upregulation of the cAMP-response element-binding protein-BDNF signaling pathway and the reduction of Aβ oligomers in the hippocampus of double-transgenic mice treated with flavanol improved rat cognitive deficits. In addition, a variety of dietary phenolic compounds have been shown to exert anti-inflammatory effects by inhibiting the secretion of cytokines such as IL-6, IL-8, and monocyte chemoattractant protein-1 in intestinal epithelial cells [40]. In general, aglycone can be easily absorbed in the small intestine, while most polyphenols are present in the form of esters, glycosides, and polymers. Flavonoid glycosides, such as rutin, which are not absorbed in the upper part of the intestine, are hydrolyzed by intestinal microorganisms in the colon. The degraded aglycone is absorbed or metabolized to phenolic acids of low molecular weights [41]. Also, phenolic acid of decomposed low molecular weight can inhibit the formation of beta amyloid aggregates and prevent neurodegenerative diseases [42]. Various plant materials that contain phenolic compounds can be used as prebiotics. According to Etxeberria *et al*. [43], polyphenol-rich sources such as green tea and wine improve the growth of bacteria in the intestines. In addition, according to Reza *et al*. [15], *Bulnesia sarmienti* (*B. sarmienti*) extract promoted the growth of *Lactobacillus* strains, and this prebiotic activity was reported to be due to the phenolic compounds contained in the *B. sarmienti* extract [15]. *Sesbania grandiflora* flower, containing rutin as a major component, promoted the growth of probiotic bacterium *L. acidophilus*, and showed that rutin content decreased in broth after 24 h incubation. This means that *L. acidophilus* grew by metabolizing rutin, a major component of *Sesbania grandiflora* flower [44]. According to Comalada *et al*. [45], quercetin glycosides reach the colon and are hydrolyzed by microorganisms, and inflammation is reduced by downregulation of the NF-κB pathway. As these reports suggest, the flavonol glycosides of AASWE might reach the colon and be degraded by microorganisms, thereby promoting the growth and activity of beneficial bacteria. Also, the aglycones and phenolic acids produced by microbes as metabolic products enhance the systemic inflammatory status through MAPK and NF-κB signaling regulation. Therefore, our results suggest that AASWE containing various phenolic compounds as antioxidants has a beneficial effect on the growth of probiotic strains and might ameliorate cognitive impairment by regulating neuroinflammation.

In conclusion, this study was conducted to examine the potential availability of AASWE as a new prebiotic and antioxidant, and to investigate the relationship between intestinal microorganism activity and LPS-induced neuroinflammation. AASWE increased the growth rate, titratable acidity and acetic acid production of *L. reuteri* strain. AASWE also showed cytoprotective effects by increasing cell viability and decreasing IL-6 expression in LPS-induced cytotoxicity in HT-29 cells. In addition, AASWE improved learning and memory function of LPS-induced cognitive deficits with the inhibition of oxidative stress, activation of the cholinergic system and regulation of pro-inflammatory mediators (*p*-JNK, *p*-NF-κB, TNF-α, and IL-6). The major bioactive compounds of AASWE that have prebiotic activity were identified as quercetin-3-*O*-arabinopyranosyl(1→2)-rhamnopyranosyl(1→6)-glucopyranose, quercetin-3-*O*-apiosyl(1→2)-galactoside, rutin, and 3-caffeoylquinic acid. Consequently, AASWE might be a useful material for improving cognitive deficits by prebiotic function by regulating neurological inflammation.

## Figures and Tables

**Table 1:** Acetic acid concentration by *Lactobacillus reuteri* strain after 48 h incubation.

Sample	Control	2% FOS	2% AASWE
Acetic acid (mM)	50.36 ± 5.38^b^	55.50 ± 1.31^b^	106.55 ± 26.18^a^

Data were analyzed using ANOVA with Duncan’s SAS and expressed as mean ± SD (*n* = 3).

Each small letter shows statistical difference and was statistically considered at *p* < 0.05.

**Table 2:** MS^2^ fragments of the identified compounds.

No.	RT (min)	*m/z* [M-H]^-^	MS^2^ fragments	Proposed compounds
1	2.35	353.08	135.04, 179.03, 191.05	3-*O*-caffeoylquinic acid
2	2.86	741.18	255.03, 271.02, 300.02	Quercetin-3-*O*-arabinopyranosyl(1→2)-rhamnopyranosyl(1→6)-glucopyranose
3	2.96	595.13	255.03, 271.02, 300.02, 301.03	Quercetin-3-*O*-apiosyl(1→2)-galactoside
4	3.07	609.14	255.03, 271.02, 300.02	Rutin
